# A cluster randomised feasibility pilot trial evaluating involving community-dwelling older adults in activities in relation to meals in a rehabilitation program; recruitment, data collection and protocol

**DOI:** 10.1186/s40814-018-0323-3

**Published:** 2018-08-06

**Authors:** M. M. Husted, A. M. Beck, L. K. Ulrikkeholm

**Affiliations:** 1 The Danish Dietetic Association, Skt. Annæ Plads 6, K 1250 Copenhagen, Denmark; 2 Copenhagen University College, Sigurdsgade 26, N 2200 Copenhagen, Denmark; 3 The Department of the Elderly and Disabled, Ørbækvej 100, SØ 5220 Odense, Denmark

**Keywords:** Rehabilitation, Meals on wheels, Dwelling older adults, Activities of daily living, Community health care

## Abstract

**Background:**

Community-dwelling older adults receiving support at home such as meals-on-wheels may lose the ability to preserve social, cognitive, and functional abilities, when becoming accustomed to and dependent of community aged care. When still able to cook older adults often hold some control over the foods that are prepared and which they eat, and which helps to foster identity. The purpose of this study is to assess feasibility of outcome measurements and sample size when conducting a pilot cluster randomized trial to evaluate community-dwelling older adults being involved in activities in relation to meals in a rehabilitation program.

**Methods:**

This cluster randomized controlled study will consist of two clusters of a total of 5 community aged care areas; the intervention cluster, which hold 3 community aged care areas and the control cluster which hold 2 areas. The 130 community-dwelling older adults, receiving meals-on-wheels, will randomly be allocated to either the intervention cluster consisting of 8 weeks of participation in a rehabilitation program led by a Case Manager or the control cluster receiving usual community aged care. The primary outcome will be assessment of data collection (ratio between completed- and non-completed data) and assessment of sample size. The secondary clinical outcomes will be health-related quality of life (EQ-5D-3 L), muscle strength (chair stand), nutritional status (weight/BMI), loneliness (UCLA scale), mental well-being (Warwich-Edinburgh scale), self-efficacy (General Self-Efficacy scale), satisfaction with food-related life (SWFL scale) and refrigerator content.

**Discussion:**

This study evaluates community-dwelling older adults receiving support at home, using involvement in activities related to meals with a rehabilitation approach, and this is a new area of research and will therefore be contributing in developing and refining consistent practices of rehabilitation programs.

**Trial registration:**

ClinicalTrials.gov (registration no: NCT03289598). The protocol has been sent to the Danish Ethical Board which has concluded that approval is not needed and that the study can be carried on as described. Approval by The Danish Data Protection Agency has been giving through general approval for use of data in The City of Odense and will follow rules for obtaining the data accordingly.

## Background

Effective and good care to older adults in the community is necessary to prevent disease, to manage chronic illness, and to stay independent as long as possible. Alongside aging come special health challenges, such as dependency, limited mobility, frailty, and other physical or mental health problems. Systematic reviews and meta-analyses suggest that nutritional support may improve clinical outcome such as mortality and complication rates [1, 2]. Only few of the studies included have been performed among community-dwelling older people receiving support at home and the major focus has been on oral nutritional supplements. With the focus of care shifting from the hospital to the community malnutrition care is to become an important issue to address in the community [3].

Studies have found a high prevalence of undernutrition among older adults receiving support at home and that this increases the risk for dependency in activities of daily living and hence the need for care [4–7]. Older adults receiving community aged care have a significant improvement in quality of life having a higher proportion of individualized activities as a rehabilitation program [8]. Rehabilitation can be defined as a series of interventions that support the individual who is at risk of impaired functioning, in achieving and maintaining the best possible functioning, including working in conjunction with the surrounding community [9].

A recent review showed that case management in community aged care can improve client psychological health or well-being and unmet service needs [10]. In addition, there is recognition that a registered dietitian, particularly one trained in self-management education techniques, may be the health care professional best-suited to deliver nutritional intervention [11, 12]. The Danish Health Authority also recommend using Dietitians as Case Managers in rehabilitation processes, since it is beneficial to have a team of professionals that are interdisciplinary as Case Managers working with rehabilitation processes in the municipalities.

Three systematic reviews have looked at benefits achieved by means of meals-on-wheels offered to older adults [13–15]. Home-delivered meal programs improve diet quality and increase nutrient intakes among participants [13]. However, more research is needed to evaluate the efficacy and effectiveness of home-delivered meals for older adults on multiple outcomes [14]. Very few randomized controlled studies have assessed the beneficial effect of meals-on-wheels as a supportive intervention [15]. In addition, no randomized controlled intervention studies have been investigating rehabilitation related to limitations of “Activities of daily living” (ADL) e.g. cooking, has any beneficial effect [16].

Support at home such as meals-on-wheels means becoming accustomed to a new mealtime experience. E.g. when still able to cook older adults often hold some control over the foods that are prepared and which they eat, and which helps to foster identity. Foods prepared at home are familiar to the person, holding memories of the past and as well, the activities of grocery shopping, meal planning, and food preparation may help to preserve social, cognitive, and functional abilities.

In this pilot trial, the primary research aim is to explore the community-dwelling older adults acceptability and feasibility of the outcome measurements as methods to measure efficacy of the intervention, and to provide data to estimate the required sample size for a future cluster randomized study of community-dwelling older adults being involved in their own meals in a rehabilitation program.

The second aim of this study is to perform a pilot cluster randomized trial to understand, on an individual level, whether community-dwelling older adults receiving meals-on-wheels experience an improvement in health-related quality of life and muscle strength, being involved in their own meals in a rehabilitation program compared to usual community aged care including receiving meals-on-wheels.

The cluster design is chosen primarily to avoid contamination, since the assigned Health Care Staff and Registered Dieticians receiving additional training could not be expected to treat individual residents differently; respectively the intervention and the control group, by preference of the older person. Also, the cluster design is chosen due to practical reasons, in order not to conflict with other nutritional projects and initiatives in the participating municipality.

## Methods

This protocol follows both the CONSORT 2010 statements extension for the reporting of cluster randomized trials [17] and the CONSORT 2010 statements extension to randomized pilot and feasibility trials [18], along with the SPIRIT 2013 guideline and checklist recommended for clinical trial protocols [19].

### Trial design

This study is a cluster randomized trial where clusters are a total of five community aged care areas in The City of Odense allocated into groups of two clusters. The two clusters are 1) the group of older adults being involved in own meals in a rehabilitation program (Intervention) (*n* = 3) and 2) usual care (Control) (*n* = 2). In the City of Odense there are 33,800 citizens above 65 years of age, and totally residents receiving support at home are 5453 (18+ years of age). 1477 citizens (18+ years of age) are receiving meals-on-wheels.

The intervention in the study consists of two parts (see below) targeted at individual participant level, and to separately investigate the two parts of the intervention a pre- and post-intervention comparison will be made.

**Fig. 1:**
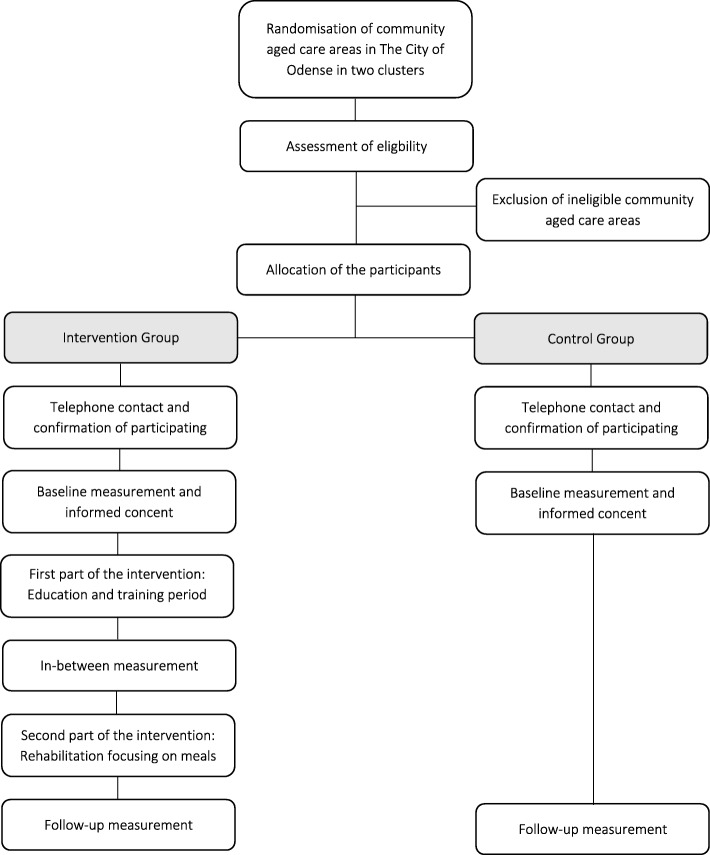
Design of the study and flow of participant

An overview of the trial is presented in Fig. 1 Design of the study and flow of participant.

### Participants

This study will use two sets of eligibility criteria, respectively for the community aged care areas (clusters) and the individual participants.

Community aged care groups (clusters): The citizens who need health care in The City of Odense are placed in a total of five community aged care areas. Excepted are those suffering from severe dementia, brain injury and mental illness, who receive special health care treatment outside the community aged care areas. All five community aged care areas will randomly be assigned into two clusters. The Intervention cluster consists of three community aged care areas: Dalum, Tagtaekker, and Skibhus, and the Control cluster consist of two community aged care areas: Rugaard, and Munkebjerg.

Inclusion and exclusion criteria for the individual participants:

- Inclusion criteria
  - Community-dwelling older adults (65+ years of age)
  - Receiving meals-on-wheels (at least one time per/week) from the municipal supplier at the time of recruitment
  - Live in The City of Odense
- Exclusion criteria
  - Moderate/severe dementia assessed by the community Health Care Staff
  - Being deaf and/or not understand the language of Danish
  - Not being able to sign the informed consent
  - Receiving (or likely to receive in the next 6 months) enteral tube feeding or parenteral nutrition;
  - Participating in other project in the municipality about nutritional support in the form of dietetic advice
  - Receiving meals-on-wheels from a private supplier at the time of recruitment
  - On an end-of-life care pathway

The reason for exclusion of older adults being deaf or not speaking Danish language is that the project has limited funding for translation into different languages, and it will not be possible to hire a deaf interpreter. It is, however, estimated by the researchers involved, that only a few will be excluded due to this reason.

### Intervention

**Fig. 2:**
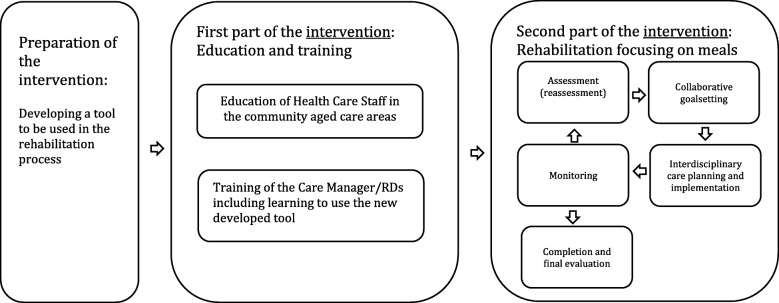
Flow of intervention, including preparation of the intervention

The entire intervention, including the phase of preparation is showed in Fig. 2 Flow of intervention, including preparation of the intervention.

In Denmark a rehabilitation process is described as short, time-bound, organized, and conducted in a holistic and interdisciplinary manner. The rehabilitation process should be built around individual goals in cooperation with the individual recipient and the professionals [20]. A rehabilitation process consists of several phases; *allocation*, *assessment*, *goalsetting*, *care planning* and *implementation*, *monitoring* and *reassessment* [10, 21]. The rehabilitation program in this study will be going through all phases of the participant’s rehabilitation after *allocated* to the study.

#### Preparation of the intervention

Based on a systematic literature research and a workshop for experts and representatives of the older adults in Denmark, a tool to be used in the rehabilitation process and the dialog between the Case Manager/RD and the older adults was developed. This tool will be new and is not a validated standardized tool. Both Goal Attainment Scaling (GAS) and “A common terminology for community aged care in Denmark” (FSIII) will be used in this tool. GAS offers many potential advantages as an outcome measure for rehabilitation, and moreover there is also evidence that GAS has positive therapeutic value in encouraging the participants to reach their goals [22]. FSIII is a generic process model using the same concept and the same way of describing the reasons for care planning and interventions in rehabilitation programs in order to efficiency improvement of the communication [23]. The tool includes the following focusses of meals:

1. manage shopping (i.e. help to plan the shopping, grocery shopping),
2. increase cooking abilities (e.g. semi-prepared meals-on-wheels, assistance to cook at home),
3. focus on social aspects of meals (e.g. eating together with family, buddies/volunteers), and
4. improve ability to eat independently (e.g. in relation to dysphagia).

The intervention consists of two parts targeted at individual participant level:

- First part of the intervention: Education and training

The first part, will consist of education of Health Care Staff in the intervention cluster starts immediately after finishing the baseline assessments and consists of a total of one day of education divided into three days, focusing of aspects of food, meals and nutrition to older adults. The education is performed by Bachelor’s Degree Nutritional and Health Educated Professionals that work within The City of Odense. Also included in the first part of the intervention, training of the Care Manager/RDs including learning to use the new developed tool was performed. The Case Managers will be Registered Dieticians (RDs) and will before commencement of the intervention be trained in managing a rehabilitation program focusing on meals. The training will consist of three days. One day as follow-up on the rehabilitation program. Specific topics include; assessment of functional abilities in relation to meals; goal setting by means of Goal Attainment Scaling (GAS) [24], ethical considerations and communication. The training program is developed specifically for the present study in co-operation with teachers from The University of Southern Denmark (SDU) - Master in Rehabilitation.

The effect of the education and training is assessed due to outcomes on an individual level (see below).

- Second part of the intervention - Rehabilitation program

In the second part of the intervention, the individual participants in the intervention cluster, will be participating in a rehabilitation program involving collaborative goal setting with a Case Manager.

Before contacting the individual participant, the Case Manager/RD will make a review of the participant’s journal in the municipalities system to find out 1) what kind of food from the meals-on-wheels is delivered, 2) if the participant presently is involved in other rehabilitation programs within the municipality, 3) the present health care situation of the participant, and 4) if the participant formerly had any visit from a RD in the municipality. These data will only be used for the rehabilitation program by the Case Manager/RD that is visiting the participant and will not be systematic collected and registered.

The *assessment* will help the participant and the Case Manager/RD to make collaborative evidence-based *goalsetting* to select initial areas for chance of meal activities.

In the present study, goals will be short-term (i.e. realistic to reach within the intervention period), and a maximum of three goals will be selected for change.

Thus, the Case Managers will encourage participants to involve in these activities.

When *implementing* the activities in relation to meals the Care Manager/RD will plan the intervention interdisciplinary with other professionals within The City of Odense e.g. Occupational Therapist, Physiotherapist, Health Care and Kitchen Staff. A period of eight weeks is chosen for the rehabilitation program in order to get a realistic comparison. This is the minimum amount of time for a Danish municipality rehabilitation program [21]. The intervention is individual and will consist of a minimum of three contacts to the Case Manager/RD in order to *monitor* and *reassess*. After completion of the rehabilitations program a final evaluation will be made.

The participants in the control group will receive usual care in relation to meals; this includes a number (one to seven) hot meals-on-wheels arriving one to three times per week, along with usual support at home.

### Outcomes

The outcome assessments, conducted in the participants homes by Research Assistants (RA) and Case Managers/RD’s will occur at inclusion (Baseline, *t* = 0) and at the end of the intervention period (Follow-up, *t* = 2). At the beginning of second part of the intervention some outcome assessments will also be conducted in only the Intervention cluster (In between, *t* = 1). It is, however, not obtained by the same person at baseline, in between, and follow-up for practical reasons. However, all will receive the same training in the methods on obtaining outcome assessments by RA (LKU) or RA (MMH). Due to the design of the study it is not possible to blind neither the RA’s nor the Case Managers/RD’s. An overview of the outcome measurements is presented in Table 1 and described in detail below.

**Table 1:** Data collection 2 Content in fridge (sufficient, insufficient, empty)

Measure	Time of data collection		
	Baseline *T* = 0	In between *T* = 1	Follow-up *T* = 2
Quality of life by EQ-5D-3 L	X	X	X
Height, meter	X	X	X
Weight, kg	X	X	X
BMI, (kg*kg/m2)	X	X	X
Muscle strength - Chair stand^1^	X	X	X
Satisfaction with food-related life	X	X	X
UCLA Loneliness Scale	X		X
The Short Warwich-Edinburgh Mental Well-being Scale	X		X
General Self Efficacy Scale	X		X
Refrigerator content^2^	X		X
Sociodemographic	X		
Meals-on-wheels (days/w)	X		

^1^Rise from a chair without using the arms in 30 s

^2^Content in fridge (sufficient, insufficient, empty)

#### Primary outcome

The choice of assessment tools is carefully selected to include factors found to be associated with nutritional intake in a former study [25] and to emphasize those factors recommended by the Danish National Board of Health to be used to evaluate community-dwelling older adults [26–28].

The feasibility of using the chosen outcome measurements will be assessed by recording the data collection by ratio between completed questionnaires/scales/record charts and non-completed or unavailable questionnaires/scales/record charts, and physical outcome measurements by ration between those participants measured and those that could not be measured.

#### Secondary outcomes

EuroQol-5D-3 L (EQ-5D-3 L) will be used to measure health related quality of life, which is the primary outcome (at *t* = 0, *t* = 1, *t* = 2). EQ-5D-3 L is a standardized instrument for use as a measure of health outcome and is recommended by the Danish National Board of Health [26–28]. The EQ-5D-3 L descriptive system comprises the following 5 dimensions (5D): Mobility, self-care, usual activities, pain/discomfort, and anxiety/depression. Each dimension has 3 levels (3 L): No problems, some problems, extreme problems. The raw score must be converted to an EQ-5D-3 L score ranging from 1.000 to − 0.624 [29]. Permission to use EQ-5D-3 L has been obtained from www.euroqol.org/.

Thirty-second chair-stand, recommended by the Danish National Board of Health [26–28] will be used to measure muscle strength (at *t* = 0, *t* = 1, *t* = 2). Participants are asked to fold their arms across the chest and to stand up and sit down on a chair without pushing off with arms, as many times as possible for 30 s. The arms may be used for assistance or for safety if need [30]. The height of the chair and mode of chair stand will be registered.

Nutritional status will be assessed by means of weight, height, and BMI (at t = 0, t = 1, t = 2). Weight (in kg to the nearest decimal) is measured (with participants wearing light indoor clothes) on calibrated project weights. As measurement of height is often not feasible in this old and frail population with chronic disease, data of height will also be retrieved from self-reported height. BMI is calculated as actual weight in kilograms divided by the square of height in meters.

Loneliness will be measured by a modified UCLA Loneliness Scale, recommended by the Danish National Board of Health [31] (at *t* = 0, *t* = 2). The scale consists of 20 items (11 positive and 9 negative), describing subjective feelings of loneliness, none of which refers specifically to loneliness. Consequently, the scale does not directly measure states that laypeople attribute as loneliness, but rather the scale measures a theoretically defined and scientifically validated understanding of loneliness. The 19 items are rated on a 4-point Likert scale in accordance with the rate of frequency, ranging from never [1] to always [4]. The 20th item in the scale; a question (How many people do you know, in fact, as you would call “my friend / my girlfriend”?), will be converted on a 4-point Likert Scale in accordance with the rate of numbers of friends, from zero friends (1), one to ten friends (2), eleven to forty-nine friends (3), and more than fifty friends (4). As described in Russel DW [32] the items 1, 5, 6, 9, 10, 15, 16, 19 and 20 will be reversed (e.g. 1 = 4, 2 = 3, 3 = 2, 4 = 1). Scores on the scale range from 20 to 80 with higher scores reflecting greater loneliness [33].

Mental well-being will be measured by the short Warwich-Edinburgh Mental Well-being Scale (SWEMWBS) – Danish version 2014 (at *t* = 0, *t* = 2). SWEMWBS is a 7-item scale; each answered on a 1 to 5 Likert scale, with most items representing aspects of psychological and eudemonic well-being, and few covering hedonic well-being or affect. Item scores are summed to produce a total score ranging from a minimum of 7 to a maximum of 49, with higher scores representing higher levels of mental well-being [34–36]. Acceptance of using the SWEMWBS Questionnaire has been obtained from Warwick Medical School, University of Warwick, UK.

Self-efficacy will be measured by The General Self-Efficacy Scale (GSE) (at *t* = 0, *t* = 2). The GSE is a 10-item psychometric scale used to assess optimistic self-beliefs to cope with a variety of difficult demands in life. A typical item is, “Thanks to my resourcefulness, I can handle unforeseen situations.” Possible responses are not at all true (1), hardly true (2), moderately true (3), and exactly true (4), yielding a total score between 10 and 40 with a higher score indicating more self-efficacy [37]. GSE is translated into Danish [38].

The satisfaction with food-related life will be measured by the Satisfaction with Food-related Life (SWFL) scale (at *t* = 0, *t* = 1, *t* = 2). The SWFL, consists of 5 items grouped into a single dimension (e.g. Food 1: Food and meals are positive elements; Food 2: I am generally pleased with my food; Food 3: My life in relation to food and meals is close to ideal; Food 4: With regards to food, the conditions of my life are excellent; Food 5: Food and meals give me satisfaction in daily life.). In each scale, the respondents must indicate their degree of agreement with these statements using a 5-level Likert scale (1 = disagree completely, 5 = agree completely). Each item will be calculated and reported separately and totally [39].

A picture of the participants’ refrigerator will be used to assess the qualitative and quantitative contents of refrigerators (at *t* = 0, *t* = 2). The categories will be “sufficient”, “in-sufficient” (e.g. with old food, according to date or appearance) or “empty” (less than 3 solid food) [40, 41].

For each participant sociodemographic data will be collected; this include age, marital status and yes/no if living alone (at t = 0). Also, data describing numbers of meals delivered by meals-on-wheels on a weekly basis will be collected.

Compliance i.e. use of and participation in possible and suggested activities in relation to meals, number of visits from Dieticians and other staffs involved, plus reasons for canceling of such planned visits and unintended adverse events/possible problems related to the suggested intervention strategies will be recorded during the 8 weeks of intervention period.

After each contact (visit or telephone) with participant following information will be registered by the Case Manager/RD:

- who were present at the meeting if the contact was a visit,
- which interdisciplinary contact within the municipality was made afterwards,
- the time spent on the specific contact,
- the Case Managers/RD’s judgment (yes/no) if the participants were motivated in the collaboration, and
- any unintended adverse events/possible problems related to the suggested intervention.

### Sample size

As this is a pilot trial the primary outcome is understanding the feasibility of implementing a large-scale trial. However, to determine sample size for sufficient power to evaluate developmental trajectories of our secondary outcome variables population estimates were derived using data from Beck et al. [42] who had the same design, population and outcome (quality of life by means of EQ-5D-3 L) as in the present study and showed a significant difference between intervention and control group in the EQ-5D-3 L follow-up score of (0.758 [0.222] versus 0.534 [0.355], (*P* = 0.001). Here it was found that the variation seen in quality of life was due to residual variation (the variation from individual to individual) and was not dependent on the clusters. Hence, with a statistical significance level of 0.05 and a power of 80% app. 53 is needed in each group. Estimating a drop-out rate of 20%, due to a longer intervention period than the former study [42], app. 130 participants are needed. This number could probably be included in approximately 10 weeks, for respectively control and intervention group.

The sample size for this trial will be compared with the actually collected samples as to estimate the required sample size for a future cluster randomized trial.

### Randomization

#### Sequence generation

A cluster randomized trial design is used, with community aged care areas, as clusters of randomization. The two clusters were randomly assigned, and it was decided at a above level of the organization in The City of Odense, not knowing about this study, which community aged care areas that should be allocated into the intervention group and the control group. The decision was made in consideration of which community aged care areas was next in having their Health Care Staff participating in the first part of the intervention; the education of the Health Care staff. The prospective participating community aged care areas will be provided with verbal information and full explanation of the trials by an RA (GBP). No written consent form is signed by the community aged care areas.

#### Allocation concealment mechanism

The allocation will be made based on clusters, which is community aged care areas, rather than individuals. A RA (LKU) from the research group will allocate the individual older adults by pre-recruitment and baseline screening, using a parallel design. The clusters will be identified before randomization of the individual participants. The cluster allocation will be going forward from a list of meals-on-wheels receivers from the municipality kitchen meeting the inclusion criteria within the clusters. Allocation concealment is not possible. After identifying the individual older person, a second RA (MMH), will contact the identified older person by phone for recruitment to the study.

#### Implementation

Initial eligibility screening will be conducted by several RA’s. First by an eligibility screen in the municipality care system, over the phone, and later in the participant’s home. One RA (LKU) will review the study protocol in detail with the potential participant and conducts an eligibility screen in the municipality care system to verify if the individual meets the inclusion or exclusion criteria. Another RA (MMH) will confirm that the participant is interested in participating in the study over the phone. If so, a time will be scheduled to visit the participant’s home. While in the home, a RA will obtain written informed consent from the participant agreeing to participate in the study. If the participant agrees to participate a comprehensive baseline assessment is then obtained to gather outcome measures.

For practical reasons, the intervention cluster will first be included during a period of 10 weeks or until the number needed according to the power calculation and then the control cluster will be included during a period of 10 weeks or until the number needed according to the power calculation.

#### Blinding

An RA from the manager group in The City of Odense performing the allocation of clusters has no contact with the community aged care groups and will be blinded from all aspects of allocation and subsequent intervention. Participants, Care Managers/RD’s, the principal investigator (MMH) and RA’s (LKU) will not be blinded for the intervention. Data will be analyzed by an external RA, who will be blinded for the results of randomization.

#### Analysis

Although this is a pilot study, we do want to explore whether or not there is a difference between groups. All statistical analysis will be performed using a statistics program (SPSS) for Windows. Data will be entered in EXCEL and will subsequently be exported into SPSS software for analysis. All participants will be included in the analysis, regardless of whether they have completed the study or not. Depending on the data type and distribution t-test, Mann-Whitney U test and Chi2 test will be used to compare changes within and between the groups. The model of data analysis needs to consider the effect of clustering; thus, this study will be using effect models with the cluster treaded as a random effect.

The intracluster correlation coefficient (ICC) for quality of life will be calculated by means of ANOVA and published to assess the appropriateness of the sample size assessment in the study.

To assess the feasibility of the outcome measurements the confidence intervals (95% CI) will be calculated to interval estimate the population.

## Discussion

When designing this present study, we had to make several considerations to overcome the challenges of preparing and design a cluster randomized trial of adequate size and quality to indicate if there is any effect of community-dwelling older adults’ involvement in activities in relation to meals in a rehabilitation program. Challenges of the complexities working with the population of community-dwelling older adults in aged care can result in underrepresentation and recruitment difficulties due to different impairments such as physical and/or cognitive problems, the consent procedure and the high attrition rates of older people participating in research [43].

### Strengths and limitations of the study

The rehabilitation approach is a new area of research in the population of community-dwelling older adults and to our knowledge, there have only been performed a few randomized controlled studies of nutritional support among community-dwelling older adults receiving support at home, using involvement in activities related to meals. This study provides an opportunity to develop and refine consistent practices of older adults’ involvement in activities in relation to meals in the communities as part of rehabilitation programs.

A clear strength of this study is the workshop in the early stage of the project, which illuminated rehabilitation due to experts, collaborators e.g. Likewise, we also consider it a strength that also representatives of the older adults in Denmark participated in this work.

In designing the study, we will choose to develop a new tool to be used in the for the rehabilitation process, but instead we could have chosen to use an already validated tool for the rehabilitation process. However, we want to make sure that the main focus of the dialog is involvement in meal related activities, and we did not find any suitable tool for this purpose.

A limitation of this study is the exclusion of severe dementia, brain injury and mental illness and this could possible reduce the representativeness of our findings. This decision is made because of the nature of the rehabilitation program including involvement in meals.

Since this study do not have much funding attached we had to make some choices in designing this study. For instance, it could have been interesting to evaluate if there would be any change in the participants community care services, use of medicine, and health challenges, since this could be an expression of the effectiveness of the intervention, however due to limitations of time and access to this information this will not be included.

## Conclusions

This protocol has defined the aims and objectives of a feasibility cluster trial and has provided a detailed description of the intervention, the study design and the methods of data collection. For the subsequent pilot trial, the protocol will be used for detail planning. The result of the subsequent pilot trial will be used to design a definitive future trial. It is expected that the results of the definitive trial will inform decisions by Registered Dieticians working with rehabilitation to involve community-dwelling older adults in activities in relation to meals, Health Care Planners working with care planning in the municipalities, and professionals planning the future rehabilitation programs for older adults.

## Strengths and limitations of this study

- This pilot study evaluates community-dwelling older adults receiving support at home, using involvement in activities related to meals with a rehabilitation approach, and this is a new area of research and will therefore be contributing in developing and refining consistent practices of rehabilitation programs.
- In planning this study, we held a workshop for experts, collaborators and representatives of the older adults in Denmark to get their opinions on the potentials of older adults’ involvement in activities in relation to meals as part of a rehabilitation program.
- A limitation of this study is the exclusion of severe dementia, brain injury and mental illness and this could possible reduce the representativeness of our findings.
- Evaluating if there would be any change in the participants community care services, use of medicine, and health challenges has been chosen not to be collected due to limitations of time and access.
